# Improving hand function in chronic incomplete tetraplegia by high-PAS intervention with alternative equipment: a case report

**DOI:** 10.1038/s41394-026-00731-7

**Published:** 2026-04-01

**Authors:** Anna Nätkynmäki, Macey Higdon, Janne Avela, Anastasia Shulga

**Affiliations:** 1 Faculty of Sport and Health Sciences, University of Jyväskylä, Jyväskylä, Finland; 2 BioMag Laboratory, HUS Diagnostic Center, Helsinki University Hospital, University of Helsinki and Aalto University School of Science, Helsinki, Finland; 3 NeuroMuscular Research Center, Faculty of Sport and Health Sciences, University of Jyväskylä, Jyväskylä, Finland

**Keywords:** Rehabilitation, Spinal cord, Spinal cord, Spinal cord injury

## Abstract

**Introduction:**

The reproducibility of a paired associative stimulation (high-PAS) protocol, shown to be beneficial for rehabilitation in incomplete spinal cord injury (SCI), was investigated in a different technical environment. Whereas previous studies relied on a specific technical setup for high-PAS, here the protocol was replicated using alternative devices. Differences in technical attributes could influence the replicability of specific output. As the necessary technology is available in many clinical and research settings, validating the protocol across device systems is important for broader implementation of high-PAS in SCI rehabilitation. In this case, a high-PAS protocol was administered over 6 consecutive weeks in a patient with severe chronic incomplete SCI.

**Case presentation:**

A 32-year-old male with chronic C5 American Spinal Injury Association Impairment Scale C tetraplegia participated in this study. Three muscle-nerve pairs from both upper extremities each received 20-min high-PAS, administered 22 times over 6 weeks. The patient was assessed by an experienced physiotherapist before high-PAS, immediately after it, and 1 month after. The intervention improved muscle strength, hand function, and daily task performance in both upper extremities.

**Discussion:**

This study sought to replicate high-PAS improvement of motor and functional scores in a patient with severe chronic incomplete SCI and demonstrated that the results can be successfully recapitulated by alternative devices. The improvement observed was consistent with previous studies and supports applicability in more versatile technical environments.

## Introduction

The physical, social, and vocational aspects of life are affected in persons with spinal cord injury (SCI) [1–3]. After injury, rehabilitation aims to restore independence and improve quality of life [1, 4]. SCI-induced functional deficits are highly heterogeneous and require individualized treatment [4–6]. In incomplete SCI (iSCI), residual neural connectivity can be strengthened to improve functional output, and neuromodulatory interventions target this connectivity to enhance recovery [7–12].

Non-invasive paired associative stimulation (PAS), which combines transcranial magnetic stimulation (TMS) over the primary motor cortex with peripheral nerve stimulation (PNS), has therapeutic potential in iSCI [7, 13, 14]. Several studies have investigated a high-intensity high-frequency variant (high-PAS), which utilizes TMS at maximal stimulator output (MSO) with high-frequency PNS trains. This specifically targets the spinal-cord level excitability and leads to the restoration of various motor functions [9, 15]. These studies included a variety of patients with iSCI 1–15 years post injury with diverse neurological levels of injury (C1-7, T7 and L1) and American Spinal Injury Association Impairment Scale (AIS) B, C, or D, based on the International Standards for Neurological Classification of Spinal Cord Injury (ISNCSCI) worksheet ©2019 [16–22]. These primary findings were replicated in 5 patients with chronic iSCI, classified as C2-5 AIS D, according to ISNCSCI, with higher age and longer post-injury, in a 4-week high-PAS intervention with a modest improvement in manual muscle test (MMT) [23]. However, the stimulation devices used in this study were essentially the same as those used in the previous studies. The same coil and navigated TMS system with incorporated EMG were used, which enabled replication of the induced electric and magnetic fields [9, 23]. In addition, the same separate PNS device with incorporated EMG was utilized, and both stimulations could be trigged for synchronized arrival, thus providing an objective and reproducible basis for defining the stimulation intensity across conditions [9, 23].

Although the technologies required for high-PAS implementation are generally considered functionally equivalent, differences in device-related attributes, such as focality, stimulation depth, threshold, and electric field characteristics, can influence the desired stimulation output when replicating previous studies to a new technical environment [24–26]. Previous high-PAS studies have relied on a specific type of system. The present study was conducted in a research environment housing different devices, used in various research and clinical settings. Based on Gutiérrez-Muto et al. [27], the TMS device used in this study falls within the most frequently used product family. The equipment used here was selected mainly because it was accessible and enabled implementation of the established high-PAS protocol. Expanding the use of high-PAS to a new technical environment is important for accessible and applicable SCI rehabilitation, as many alternative devices exist in various research and clinical environments. This case study sought to determine whether the effects of high-PAS could be replicated in a technical environment that differs considerably from previous high-PAS studies, and to explore the applicability in a patient with severe chronic iSCI and a clear disparity in hand function [9, 16, 28, 29].

### Case presentation

A 32-year-old male with incomplete tetraplegia, 5 years post-injury (2018), participated in the study. Based on ISNCSCI exam, he had a C5 AIS C injury, with a clear disparity in hand function. He was informed of his right to withdraw from the study at will. The study was approved by the Ethics Committee of the Hospital District of Helsinki and Uusimaa.

**Fig. 1:**
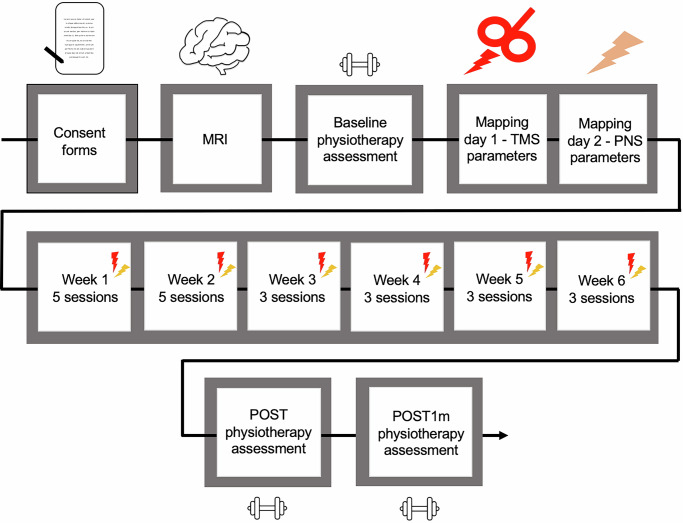
Study structure. The study proceeded through consent forms, MRI, and a physiotherapy assessment, followed by two mapping days, a 6-week intervention period, and physiotherapy evaluations immediately after the intervention and 1 month later. MRI magnetic resonance imaging, TMS transcranial magnetic stimulation, PNS peripheral nerve stimulation.

Before the intervention, the patient signed consent forms, and underwent a physiotherapy assessment. Magnetic resonance image (MRI) for navigated TMS was obtained. Prior to the intervention, the patient had been receiving long-term physiotherapy at a frequency of approximately two sessions per week, focusing on manual therapy, active training of core strength, posture, and balance. Since the high-PAS sessions were initially more frequent, he decided to reduce the physiotherapy sessions to once per week. However, after the initial phase, he returned to the pre-intervention schedule. He had no medication that affected the central nervous system during the study. The study structure is presented in Fig. 1.

An experienced physiotherapist, blinded to the experimental setup, assessed performance before (Baseline), immediately post-intervention (POST) and at the 1-month follow-up (POST1m). Outcome measures included MMT, the ISNCSCI worksheet for upper extremities, the Spinal Cord Independence Measure (SCIM), several hand function tests, selected activities of daily living (ADL), spasticity assessment (MAS), and pain questionnaire. A summary of all assessments is provided in Table 1.

**Table 1:** Assessments performed by the physiotherapist.

**Assessment**		**Information**	**Scoring**
Manual Muscle Test (MMT)		Total scores for all 37 evaluated muscles and scores for all 25 muscles innervated by each of the stimulated nerves. For more, see Hislop et al. [39].	0–5; 0 = complete lack of voluntary muscle contraction, examiner unable to feel or see any muscle contraction; 5 = able to complete full range of motion and maintain end point range position against maximal resistance.
ISNCSCI Worksheet © 2019		Sensory levels (C2-T2 dermatomes for key sensory points) and motor levels (C5-T1 key muscles) for upper extremities. For more, see Rupp et al. [31].	(0–5, NT) motor; 0 = total paralysis, 5 = (normal) active movement, NT not testable.
			(0–2, NT) sensory; 0 = absent, 1 = altered, 2 = normal, NT not testable.
The Spinal Cord Independence Measure (SCIM)		The ability of patients with spinal cord lesions to perform everyday tasks according to their value for the patient. For example, see Catz & Itzkovich 2007 [40].	(0–20) self-care; (0–40) respiration and sphincter management; (0–40) mobility.
Hand function tests	Box and block	Unilateral gross manual dexterity. For example, see Mathiowetz et al. 1985a [41].	0–150
	9-hole-peg	Finger dexterity. For example, see Mathiowetz et al. 1985b [42].	0–18
	Grip strength	Hand dynamometer (Jamar Plus+ Digital Hand Dynamometer, Performance Health Supply Inc., USA), grip width 3 for males, 3 attempts, highest recorded. For example, see Mathiowetz et al. 1985c [43].	Metric, based on execution.
	Pinch strength	Pinch gauge (Baseline^®^ Mechanical Pinch Gauge, Fabrication Enterprises Inc., USA), index and thumb pinch, key pinch and three-finger pinch tasks, 2 attempts, higher recorded. For example, see Mathiowetz et al. 1985c [43].	Metric, based on execution.
Activity of daily living (ADL)		The ability to perform 10 functional tasks in daily life. Custom made test. See Table 4 for results and for more, see Shulga et al. [33].	0–1; 0 = not able to perform task, 1 = able to perform task.
Modified Ashworth Scale (MAS)		Spasticity of upper extremities. For more, see Ashworth Spasticity Scale (and Modified Version) [44].	0–4; 0 = no increase in tone, 4 = limb rigid in flexion or extension.
International Spinal Cord Injury Pain Basic Data Set, version 3.0		Standardized method to assess specific pain complications as well as multiple pain problems. For more, see Biering-Sørensen et al. [45].	(Yes/no) if pain; (0–5) 1 question assessing pain issues; (0–10) 3 questions assessing pain interference; assessment of pain places and type; (0–10) magnitude; (yes/no) use of treatment.

Baseline assessment values represent the patient’s physical status prior to the intervention. For Baseline MMT (0–5 scale), the average score of all upper extremity muscles was 1.9 in the right and 2.9 in the left. Thirty-seven muscles per upper extremity were evaluated; 21 muscles on the right and six on the left scored 0. The SCIM at Baseline was 57/100. According to the SCIM mobility subscale, he was classified as a manual wheelchair user who was independent indoors and at moderate distances but required partial assistance for longer outdoor distances. He was also capable of maintaining 2–3 activities in bed and preventing pressure injuries without electric aids, and being able to transfer with assistance. According to the SCIM self-care subscale, he was independent in feeding and grooming. For bathing, he was independent with adaptive devices or in a specific setting. For dressing, he was independent with clothing without buttons, zippers, or laces.

The precise methods and technical environment used in the previous studies are reviewed in Shulga et al. [9]. The muscles and their innervating nerves targeted in the study were abductor pollicis brevis (APB) and median nerve, abductor digiti minimi (ADM) and ulnar nerve, and extensor digitorum communis (EDC) and radial nerve on both upper extremities. To individually optimize high-PAS for the patient, the first mapping day was used to determine TMS parameters, such as optimal stimulation sites, resting motor thresholds (rMT), and motor-evoked potential (MEP) latencies. The second day was used to define PNS parameters, including F-response latency and intensity [19, 29, 30]. Both latencies are required to accurately determine optimal interstimulus interval (ISI) for each muscle-nerve pair. The mapping results are presented in Table 2.

**Table 2:** Mapping results for both extremities’ muscle-nerve pairs.

Side	Muscle	Nerve	rMT % MSO	MEP latency (ms)	F-response latency (ms)	PNS intensity (mA)	ISI for high-PAS (ms)	Preactivation or motor imagery
Right	APB	Median	Over 100	34.5	32	4	−2	Opposition of I-II-III fingers
	ADM	Ulnar	Over 100	36	33	12.5	−3	Flexion of IV-V fingers
	EDC	Radial	48	18	12	6	−6	Wrist extension
Left	APB	Median	60	32	33.2	5	1 (1.2 fault)	Opposition of I-II-III fingers
	ADM	Ulnar	68	36.5	34.5	9.5	−2	Flexion of IV-V fingers
	EDC	Radial	48	21.4	16	28	−5	Wrist extension

With right APB and ADM, the patient was instructed to imagine muscle contraction. ISI within the range of −1–1 could only be executed with an accuracy of “as close as possible” in the Spike2 software when monitoring the stimulation sequences. By assessing the 1-ms ISI difference between TMS and PNS pulses from recorded offline traces, the timing error was found to be 0.2 ms and was accepted for use in the intervention.

Twenty-two high-PAS sessions were distributed over 6 consecutive weeks. In each high-PAS session, both upper extremities of the patient were stimulated in a randomized order. Since one muscle-nerve pair was stimulated for 20 min, one complete stimulation session with three pairs from both extremities took approximately 2 h 40 min, including breaks. During the initial stimulations, the patient was seated in the MagVenture chair, seating was changed to his own wheelchair for comfort reasons. Additionally, a neck support attachment was built to support this position. Accurate coil position and the patient were constantly monitored by two researchers throughout the sessions to detect coil shifts and possible side effects such as autonomic dysreflexia. Stimulations were coupled with preactivation or motor imagery (if the patient was not capable of performing the movement), instructed to start immediately before the pulse and end right after it, to ensure adequate relaxation between pulses.

**Fig. 2:**
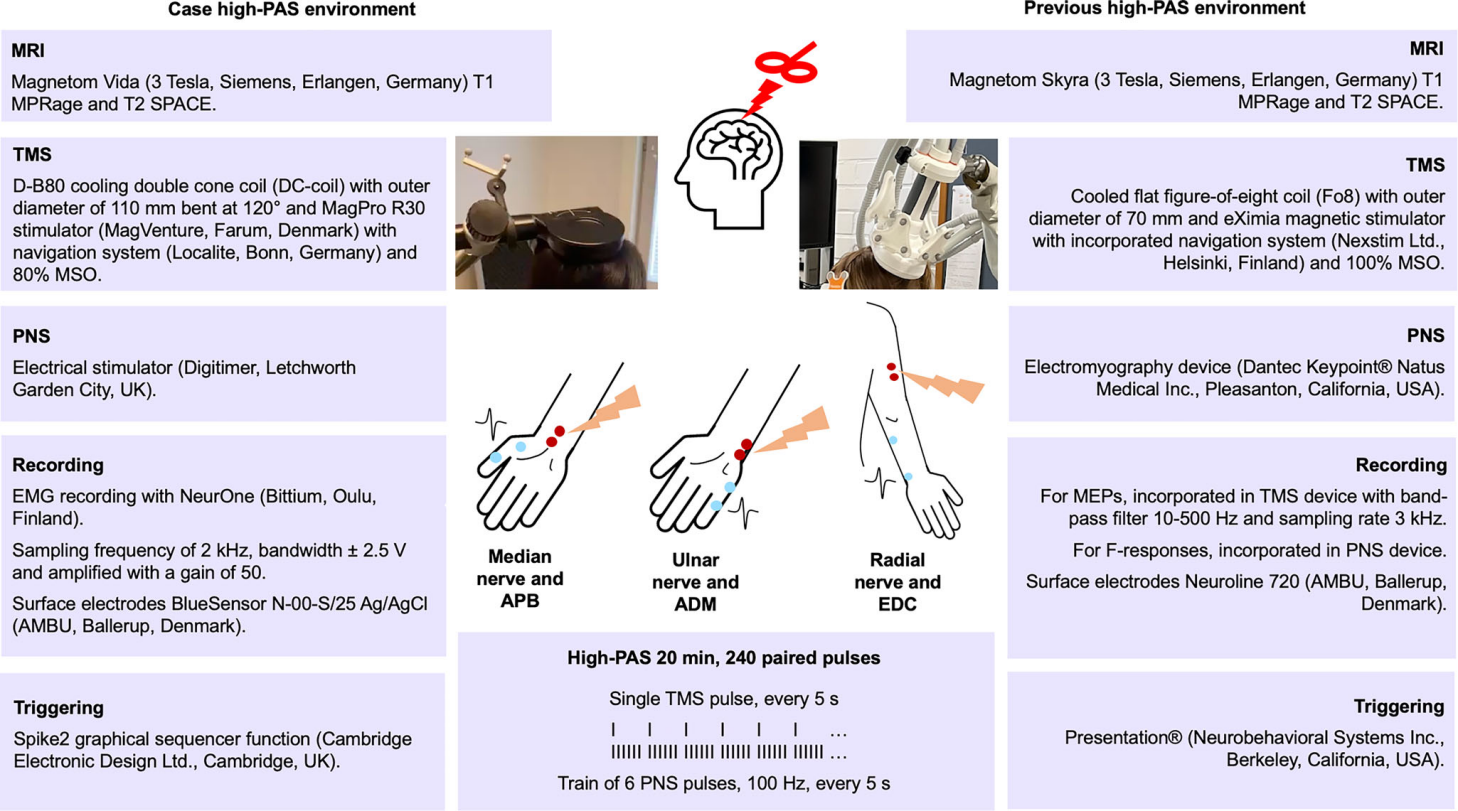
Devices used in high-PAS of the current case and in previous studies. Navigated high-intensity single pulse TMS was directed to the right motor cortex time-locked with the PNS on the left periphery and vice versa to the other side for spinal-cord level targeting. All TMS stimulations were targeted to correct hotspots over primary motor cortex using the reconstructed head model from MRIs. High-frequency PNS train of six pulses (100 Hz) was delivered to a peripheral nerve (lightning and two proximal red electrodes; median nerve at palmar wrist at carpal tunnel; ulnar nerve at the wrist proximally to Guyon canal; radial nerve proximally to lateral epicondyle with pressure to maintain contact due to nature of the limb tissue). MEPs were measured from selected muscles with surface EMG (wave response and two distal blue electrodes; APB muscle belly and distal side of 1^st^ metacarpophalangeal joint; ADM muscle belly and distal side of 5^th^ metacarpophalangeal joint; EDC muscle belly and styloid process of ulna). Replicating the original protocol, high-PAS for a single muscle-nerve pair consisted of 240 paired pulses of TMS and PNS with 5-s interval at 0.2 Hz frequency between consecutive paired pulses.

The devices used in previous high-PAS studies and the current case are presented in Fig. 2. However, despite changes in device systems, both environments provided essential tools for close replication and successful implementation of the high-PAS protocol. Both implementations adhered to their available navigation tools for consistent TMS-induced magnetic fields over the desired sites throughout the weeks of intervention. TMS pulses and PNS trains were successfully triggered to ensure consistent spinal-cord level targeting.

Because device-related attributes differed considerably between technical environments of the present experiment and previous studies, the setups were not comparable, and an identical replication was not possible. Therefore, replicating as closely matched output as possible required precise adjustments. A major difference was that the cooled double-cone coil (DC coil) and stimulator used in current case produced different fields and related characteristics than the cooled flat figure-of-eight (Fo8) coil and stimulator used in the previous high-PAS studies. Therefore, pilot measurements were conducted with four healthy participants (mean age 29 ± 2 years, 1 male). The rMT for the non-dominant left ADM ranged between 27–35% MSO. TMS with 80% MSO intensity was selected for high-PAS. MEP amplitudes measured before, immediately after, and 30 and 60 min after a 20-min high-PAS were compared to evaluate the efficacy of 80% MSO stimulation intensity for case intervention. At the group level, MEPs measured immediately after high-PAS increased by 101% (+ 307 µV), by 57% (+ 96 µV) at 30 min after, and by 106% (+ 690 µV) at 60 min after. Thus, the intensity of 80% MSO was chosen for the intervention to be sure that we do not exceed the previously used TMS intensity.

**Fig. 3:**
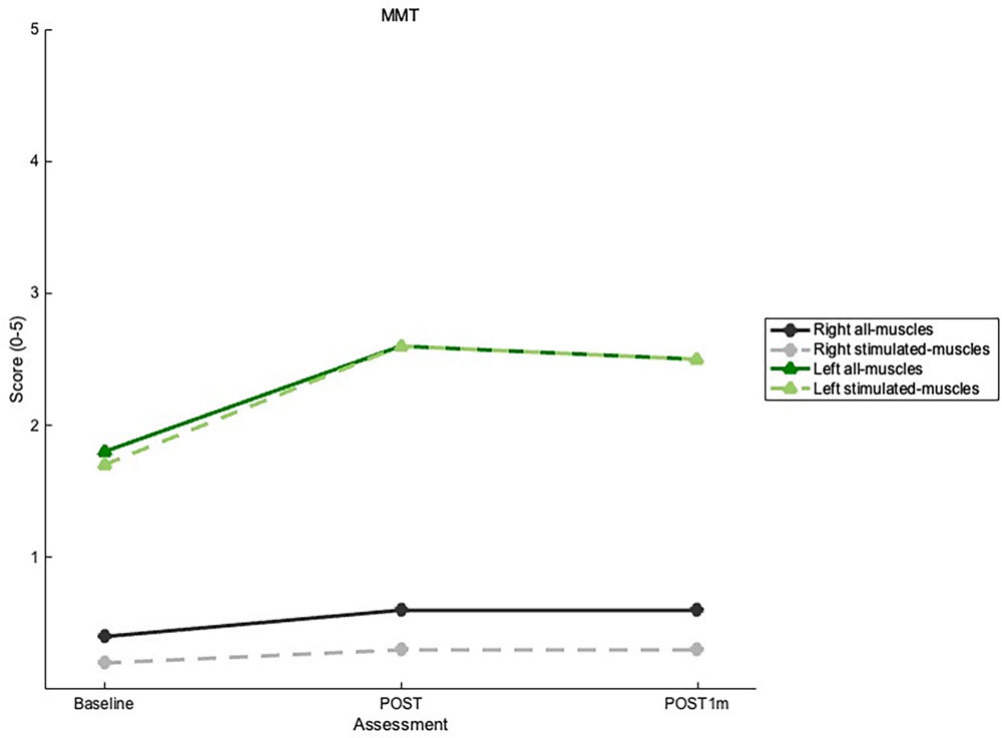
MMT results from Baseline, POST, and POST1m according to all-muscles (37) evaluated and stimulated-muscles (25) evaluated per upper extremity. Each muscle that scored 5 at Baseline was excluded to assess improvement in only the affected muscles.

The main outcome, MMT, was measured on all 37 muscles (all-muscles) from both upper extremities, including the 25 muscles innervated by the three nerves stimulated (stimulated-muscles), which were analyzed separately (Table 3). MMT strength score of all-muscles and stimulated-muscles improved on both extremities (Fig. 3). This indicates that strength improved in some muscles that were not directly stimulated (e.g. pectoralis major, subscapularis, and teres major). Spasticity and pain remained at score 0.

**Table 3:**
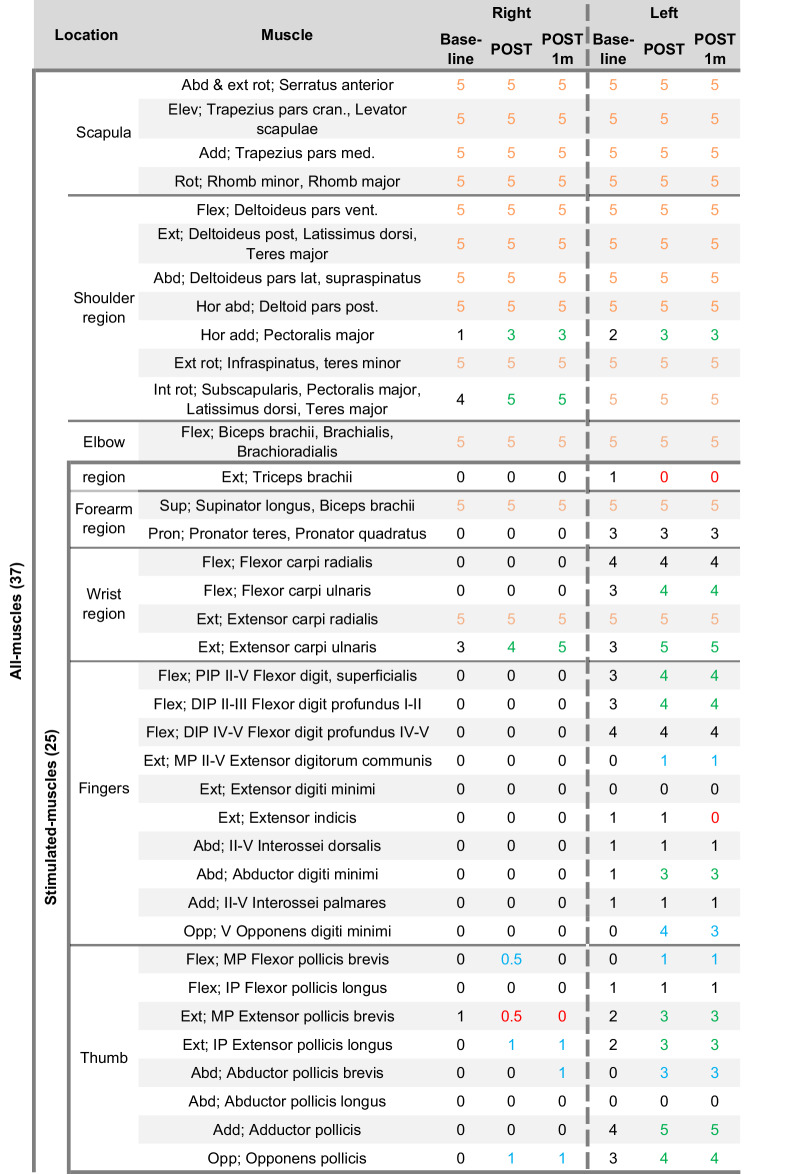
MMT results according to every muscle evaluated per timepoint.

*Abd* abduction, *Add* adduction, *Elev* elevation, *Ext* extension, *Flex* flexion, *Hor* horizontal, *Int* internal, *Opp* opposition, *Pron* pronation, *Rot* rotation, *Sup* supination.

Scoring; 0 = complete lack of voluntary contraction. The examiner is unable to feel or see any muscle contraction. 1 = Faint or “flicker” muscle contraction without any movement of the limb. The examiner can see or palpate some contractile activity of the muscle/s or may be able to see or feel the tendon “pop up” or tense as the person tries to perform the contraction. 2 = Very weak muscle contraction with movement through complete range of motion in a position that eliminates or minimizes the force of gravity. 3 = Muscle can complete full range of motion against only the resistance of gravity. 4 = Able to complete the full range of motion against gravity and can tolerate strong resistance without breaking the test position. 5 = Able to complete full range of motion and maintain end point range position against maximal resistance. Orange indicates the muscles scoring 5 at Baseline and excluded from the analysis. Green is positive change from Baseline. Red is negative change from Baseline. Muscles that reverted from 0–1 or more (i.e. gained movement from full paralysis), are marked in blue.

SCIM, ISNCSCI worksheet, Hand function, and ADL scores of the physiotherapy assessments are presented in Table 4. Relevant to motor abilities, scores in the mobility subsection of SCIM improved in transfer actions and in action to prevent pressure injuries. For the left side, the ISNCSCI motor score decreased at POST, but increased at POST1m. The right-side score remained unchanged; and the sensory scores varied for both sides. Most hand function results fluctuated, but notable increases were found in the right box and block score and in the left grip strength. For the right side, the patient acquired three new daily living activity tasks. The capability of the left side remained the same.

**Table 4:** Physiotherapy assessment results of SCIM, ISNCSCI worksheet, Hand function, and ADL.

*LT* light touch, *PP* pin prick, *Green* positive change from Baseline, *Red* negative change from Baseline.

## Discussion

The present study demonstrates that the high-PAS protocol, as reviewed by Shulga et al. [9], can produce comparable improvements in a patient with a chronic severe case of iSCI when adapted to an alternative technical environment. Despite differences in devices, the intervention in the current study led to measurable improvements. The considerable functional disparity of the patient’s hands was reflected in the results. Importantly, the patient acquired new, functionally meaningful daily skills, such as cutting bread, taking an object from a diagonal position, and opening a lock (bathroom door). Specifically, high-PAS facilitated restoration of volitional contractions in a few previously paralyzed muscles and enhanced strength in several weakened ones.

Disparity was reflected in the increases of MMT scores of stimulated-muscles averaging 0.7 in the left and 0.2 in the right extremity. Initially, the patient could complete all hand function tests with the left, while only the box and block test succeeded with the right extremity. After the intervention, muscle force and fine motor dexterity improved in the left, and gains in coarse movements involving the thumb were observed in the right extremity. Recovery of volitional activity in four thumb muscles, initially scoring 0, likely explains the gains observed in box and block and ADL scores. A longer intervention might have led to further improvements [16, 18]. MMT alone may be less sensitive in detecting subtle changes in the more affected right extremity, but hand function tests complemented the results by capturing functional improvements.

Several studies have reported improved MMT scores following high-PAS in patients with iSCI. Average increases of 1 [18] and 1.4 points [20] in upper extremities, and 1.2 points [17] in lower extremities, have been observed after an intervention lasting 4 weeks to a few months. In study where high-PAS continued as long as MMT scores improved, gains by 3.2 points for the weaker left and 1.3 for the stronger right upper extremity after 47 weeks of high-PAS have been reported [16]. In contrast, our patient showed smaller average increases by 0.2 points (POST) and 0.3 points (POST1m) on the right and by 0.8 points (POST and POST1m) on the left upper extremity, which were lower than previously reported results. This may reflect the patient’s relatively severe iSCI, potentially influenced by physiological factors such as the severity of upper and lower motor neuron lesions, the shorter intervention period, and differences in technical device parameters. Other high-PAS studies suggest that less severe impairments may benefit from shorter interventions, and residual muscle strength is positively associated with high-PAS outcomes [9, 16–18, 20, 31].

Patients in previous high-PAS studies maintained consistent behavior habits throughout interventions. Here, weekly rehabilitation sessions were temporarily reduced according to the patient’s preference, which differed from prior protocols. Importantly, the rehabilitation program depends on the patient’s injury and state and therefore must be individualized [32]. Given the patient’s overall wellbeing and ability to manage stimulations, this reduction during the initial phase of the intervention likely had minimal impact on the observed improvements. In the current study, the patient reported greater ease of use of the left hand after the intervention. While learning effects cannot be fully excluded, this likely reflects improvements in SCIM mobility and ADL scores [33]. Enhanced muscle activation and force generation may support better performance in daily activities, as even small strength gains can positively impact everyday life [9]. A slight, non-systematic increase in key sensory detection was observed, although sensory outcomes have generally remained stable in prior high-PAS studies [16–19, 21, 33]

The MEPs were difficult to elicit from the right APB and ADM, likely reflecting more severe neuronal denervation in the right extremity [34]. The severity of the lower motor neuron lesion, as assessed by motor point integrity test, correlates with MMT outcomes and could confirm the extent of denervation and validate the findings [34]. Denervation severity may be a critical consideration in influencing the efficacy of high-PAS, but in this case the intervention improved function of both extremities.

This study supports the use of a high-PAS protocol across various technical environments. However, several factors must be considered when implementing high-PAS in new settings. One key factor is the combination of TMS coil and stimulator. The peak electric field strength of the coil is inversely related to the motor threshold, which is typically expressed as a percentage of MSO and is influenced by the peak electric field strength, pulse waveform, and maximum stimulator-specific strength [35]. Schecklmann et al. [26] reported that the DC coil produces a higher magnetic field in deeper brain regions and reduces rMT compared with the Fo8 coil of the same device [26]. However, the DC coil offers greater benefits for deeper leg areas, whereas its advantages are less pronounced for superficial hand regions. In contrast, the Fo8 coil, like the coil used in previous high-PAS studies, is reported to have a more favorable depth-focality tradeoff [25]. The rationale for using 100% MSO in high-PAS interventions is to generate repeatable and reliable orthodromic volleys in the corticospinal tract – and given that the DC coil is reported to induce a higher magnetic field than the Fo8 coil with comparable setups, no conclusions about device superiority can be drawn from a single patient in this context [9, 26].

We searched for the highest TMS intensity that would not exceed the strength of the previously employed 100% MSO and would not lead to the discomfort often associated with a DB coil compared with a flat Fo8 [24]. Based on our previous experience, strong TMS intensity can induce body sway and facial muscle twitches, which may lead to distress, disruption of ergonomic position, and a decrease in coil location control. Besides the initial seating, no adverse effects were observed during this study.

The adequacy of the selected stimulation intensity is supported by the average rMT calculated in the pilot measurements. In healthy pilot participants, rMT ranged between 27–35% (mean 31%) MSO when using the DB coil. Cortes et al. [36] reported rMTs for the flexor carpi radialis and extensor carpi radialis muscles using a MagVenture stimulator and D-B80 coil, including 10 chronic cervical SCI patients and healthy controls [36]. Patients with SCI had rMTs ranging between 38–80% (mean 52%) MSO, while controls averaged 32% MSO. Higher rMTs in SCI patients are well established [37]. Similarly, Fassett et al. [37] found rMTs exceeding 60% MSO in 9 patients with chronic SCI patients for three hand muscles compared with an approximation of 50% MSO in matched healthy controls using the Fo8 coil and a Magstim Plus stimulator, consistent with our results [37]. Comparing induced field strength in the cortex between the experimental setups might be an additional useful means to evaluate the equivalence of the selected stimulation parameters between different TMS devices.

Although this study was limited to a single subject with no controls, the results suggest that high-PAS can lead to functional improvements in an individual with chronic severe iSCI when applied using the reported setting and stimulation devices. Notably, while all previous studies employed essentially the same devices, the current study used a different setup combination. Despite this variation, the observed improvements indicate that the high-PAS methodology may remain effective across different device systems. Nonetheless, it is not possible to definitively determine the most suitable equipment. It should be noted that a study of a single patient without control does not indicate the superiority or inferiority of different device setups. Further research incorporating different device systems and diverse patient profiles is needed for broader applicability of the intervention.

Spinally targeted high-PAS modulates excitability at the spinal-cord level within the sensorimotor system, potentially strengthening connectivity and supporting hand rehabilitation after SCI [9, 15, 16, 38]. The devices required for the implementation of the high-PAS protocol are already available in several research-based and clinical environments, and daily stimulation sessions can be administered by specifically trained personnel. Therefore, high-PAS could feasibly and effectively be implemented in clinical practice to improve the independence and quality of life for patients with SCI.

## Data Availability

The datasets generated during and/or analyzed during the current study are available from the corresponding author on reasonable request.
